# Supplementary material for: Pyruvate Dehydrogenase Kinase 1 inhibition mediated oxidative phosphorylation enhancement in cartilage promotes osteoarthritis progression
Source: BMC Musculoskelet Disord. 2023 Jul 20;24:597. doi: 10.1186/s12891-023-06585-6 (PMC10357736; doi:10.1186/s12891-023-06585-6)
Supplement: Supplementary file 1 — Additional file 1. [file 12891_2023_6585_MOESM1_ESM.zip › KEGG.Gsea.1653622432464/KEGG_THYROID_CANCER.html]

Details for gene set KEGG\_THYROID\_CANCER[GSEA]

|  || Dataset | 55457\_collapsed\_to\_symbols.cls.cls #OA\_versus\_normal.cls.cls #OA\_versus\_normal\_repos |
| Phenotype | cls.cls#OA\_versus\_normal\_repos |
| Upregulated in class | normal |
| GeneSet | KEGG\_THYROID\_CANCER |
| Enrichment Score (ES) | -0.37840292 |
| Normalized Enrichment Score (NES) | -1.2853795 |
| Nominal p-value | 0.13793103 |
| FDR q-value | 0.3067678 |
| FWER p-Value | 1.0 |
Table: GSEA Results Summary

  

Fig 1: Enrichment plot: KEGG\_THYROID\_CANCER      
 Profile of the Running ES Score & Positions of GeneSet Members on the Rank Ordered List

  

| SYMBOL | TITLE | RANK IN GENE LIST | RANK METRIC SCORE | RUNNING ES | CORE ENRICHMENT || 1 | MAPK3 | mitogen-activated protein kinase 3 [Source:HGNC Symbol;Acc:HGNC:6877] | 757 | 0.579 | 0.0114 | No |
| 2 | CDH1 | cadherin 1 [Source:HGNC Symbol;Acc:HGNC:1748] | 2589 | 0.292 | -0.0954 | No |
| 3 | NTRK1 | neurotrophic receptor tyrosine kinase 1 [Source:HGNC Symbol;Acc:HGNC:8031] | 3015 | 0.248 | -0.0983 | No |
| 4 | TPM3 | tropomyosin 3 [Source:HGNC Symbol;Acc:HGNC:12012] | 3484 | 0.204 | -0.1099 | No |
| 5 | PAX8 | paired box 8 [Source:HGNC Symbol;Acc:HGNC:8622] | 3680 | 0.187 | -0.1023 | No |
| 6 | TFG | trafficking from ER to golgi regulator [Source:HGNC Symbol;Acc:HGNC:11758] | 3719 | 0.183 | -0.0830 | No |
| 7 | TCF7L2 | transcription factor 7 like 2 [Source:HGNC Symbol;Acc:HGNC:11641] | 5034 | 0.070 | -0.1766 | No |
| 8 | TP53 | tumor protein p53 [Source:HGNC Symbol;Acc:HGNC:11998] | 5511 | 0.032 | -0.2097 | No |
| 9 | TPR | "translocated promoter region, nuclear basket protein [Source:HGNC Symbol;Acc:HGNC:12017]" | 5919 | 0.001 | -0.2412 | No |
| 10 | PPARG | peroxisome proliferator activated receptor gamma [Source:HGNC Symbol;Acc:HGNC:9236] | 6006 | -0.005 | -0.2473 | No |
| 11 | MAPK1 | mitogen-activated protein kinase 1 [Source:HGNC Symbol;Acc:HGNC:6871] | 6853 | -0.068 | -0.3049 | No |
| 12 | RXRG | retinoid X receptor gamma [Source:HGNC Symbol;Acc:HGNC:10479] | 6947 | -0.075 | -0.3029 | No |
| 13 | NRAS | "NRAS proto-oncogene, GTPase [Source:HGNC Symbol;Acc:HGNC:7989]" | 7300 | -0.102 | -0.3179 | No |
| 14 | RXRA | retinoid X receptor alpha [Source:HGNC Symbol;Acc:HGNC:10477] | 7381 | -0.109 | -0.3109 | No |
| 15 | TCF7L1 | transcription factor 7 like 1 [Source:HGNC Symbol;Acc:HGNC:11640] | 7383 | -0.109 | -0.2977 | No |
| 16 | LEF1 | lymphoid enhancer binding factor 1 [Source:HGNC Symbol;Acc:HGNC:6551] | 7467 | -0.116 | -0.2901 | No |
| 17 | CCND1 | cyclin D1 [Source:HGNC Symbol;Acc:HGNC:1582] | 7512 | -0.119 | -0.2791 | No |
| 18 | HRAS | "HRas proto-oncogene, GTPase [Source:HGNC Symbol;Acc:HGNC:5173]" | 7977 | -0.153 | -0.2965 | No |
| 19 | BRAF | "B-Raf proto-oncogene, serine/threonine kinase [Source:HGNC Symbol;Acc:HGNC:1097]" | 8171 | -0.169 | -0.2910 | No |
| 20 | RET | ret proto-oncogene [Source:HGNC Symbol;Acc:HGNC:9967] | 9297 | -0.256 | -0.3474 | Yes |
| 21 | NCOA4 | nuclear receptor coactivator 4 [Source:HGNC Symbol;Acc:HGNC:7671] | 9589 | -0.282 | -0.3357 | Yes |
| 22 | MAP2K2 | mitogen-activated protein kinase kinase 2 [Source:HGNC Symbol;Acc:HGNC:6842] | 9932 | -0.315 | -0.3241 | Yes |
| 23 | MAP2K1 | mitogen-activated protein kinase kinase 1 [Source:HGNC Symbol;Acc:HGNC:6840] | 10197 | -0.340 | -0.3033 | Yes |
| 24 | KRAS | "KRAS proto-oncogene, GTPase [Source:HGNC Symbol;Acc:HGNC:6407]" | 10544 | -0.377 | -0.2844 | Yes |
| 25 | CCDC6 | coiled-coil domain containing 6 [Source:HGNC Symbol;Acc:HGNC:18782] | 10570 | -0.381 | -0.2401 | Yes |
| 26 | RXRB | retinoid X receptor beta [Source:HGNC Symbol;Acc:HGNC:10478] | 11871 | -0.565 | -0.2725 | Yes |
| 27 | TCF7 | transcription factor 7 [Source:HGNC Symbol;Acc:HGNC:11639] | 12628 | -0.813 | -0.2326 | Yes |
| 28 | MYC | "MYC proto-oncogene, bHLH transcription factor [Source:HGNC Symbol;Acc:HGNC:7553]" | 12801 | -0.970 | -0.1283 | Yes |
| 29 | CTNNB1 | catenin beta 1 [Source:HGNC Symbol;Acc:HGNC:2514] | 12866 | -1.119 | 0.0026 | Yes |
Table: GSEA details [plain text format]

  

Fig 2: KEGG\_THYROID\_CANCER      
 Blue-Pink O' Gram in the Space of the Analyzed GeneSet

  

Fig 3: KEGG\_THYROID\_CANCER: Random ES distribution      
 Gene set null distribution of ES for **KEGG\_THYROID\_CANCER**
